# *In situ* synthesis of MWCNT-graft-polyimides: thermal stability, mechanical property and thermal conductivity

**DOI:** 10.1039/d0ra00449a

**Published:** 2020-04-02

**Authors:** Chunbo Wang, Bing Cong, Junyu Zhao, Xiaogang Zhao, Daming Wang, Hongwei Zhou, Chunhai Chen

**Affiliations:** Key Laboratory of High Performance Plastics (Jilin University), Ministry of Education, National & Local Joint Engineering Laboratory for Synthesis Technology of High Performance Polymer, College of Chemistry, Jilin University Changchun 130012 P. R. China cch@jlu.edu.cn

## Abstract

Herein, MWCNT-graft-polyimides (MWCNT-*g*-PIs) were prepared by the *in situ* grafting method. Strengthening the interfacial interaction between MWCNTs and polyimide chains decreased their interfacial thermal resistance (*R*_C_). In contrast to the *R*_C_ of 10% MWCNT/PIs, the *R*_C_ of 10% MWCNT-*g*-PI decreased by 16.7%. Hence, MWCNT-*g*-PIs possessed higher thermal conductivity than MWCNT/polyimides (MWCNT/PIs). Meanwhile, the *T*_g_ values of all the samples (MWCNT/PIs and MWCNT-*g*-PIs) were greater than 399 °C (by DMA). Compared with MWCNT/PIs, 5% and 10% MWCNT-*g*-PIs showed enhancement in thermal stability in air. The storage modulus retentions were greater than 63% at 200 °C and 45% at 300 °C. Also, 5% and 10% MWCNT-*g*-PIs maintained the high tensile strength of pure PI, and the tensile modulus increased up to 2.59 GPa on increasing the loading amount of MWCNTs. This study sheds light on improving the thermal conductivity of polyimides effectively at relatively low loadings.

## Abstract 2

In situ synthesis of MWCNT-graft-polyimides enhanced thermal conductivity at a relatively low loading.

## Graphical abstract

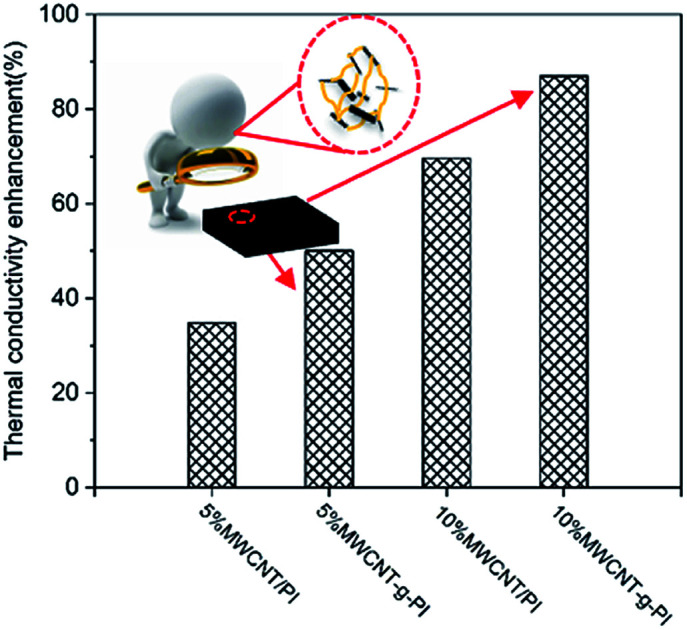

## 1. Introduction

In recent years, with the rapid development of high-performance microelectronic equipment and energy harvesting devices, the demand for heat sinks in industrial and electronic fields has dramatically increased.^1,2^ However, the thermal conductivity of common polymers is quite low and ranges from 0.1 W m^−1^ K^−1^ to 0.3 W m^−1^ K^−1^. Hence, their applications are severely limited in industrial and electronic fields due to heat accumulation.^3–5^

It is important to increase the thermal conductivity of polymers to enhance the thermal diffusion and then reduce the heat accumulation. A simple and feasible method for enhancing the thermal conductivity of polymers involves introducing highly thermally conductive fillers (carbon nanotubes,^4,6,7^ graphites,^8–10^ boron nitrides,^11,12^ aluminum nitrides,^13,14^ and aluminum oxides^15^) into polymers.

Among all kinds of highly thermally conductive fillers, carbon nanotubes (MWCNTs or SWCNTs) have been expected to be capable of improving the thermal conductivity of polymers effectively at relatively low loadings.^16–19^ However, the poor thermal conductive performances of carbon nanotube composites are due to the high interfacial thermal resistance between carbon nanotubes and polymers.

Improving the filler/polymer interfaces can reduce “thermal resistance”, and some methods have also been considered, such as non-covalent functionalization^7^ and covalent functionalization.^6^ Covalent functionalization involves grafting some chemical functional groups (amines, silanes, polymers, *etc.*) onto carbon nanotubes.

In this paper, polyimide was selected as a polymer matrix owing to its outstanding thermal and mechanical properties and MWCNTs acted as thermally conductive fillers. MWCNT-graft-polyimides (MWCNT-*g*-PIs) were obtained by the *in situ* grafting method for reducing the interfacial thermal resistance between nanotubes and polyimide to enhance the thermal conductivity. The thermal stability, mechanical properties and thermal conductivity of MWCNT-*g*-PIs were studied. For comparison, MWCNT/polyimides (MWCNT/PIs) were prepared by a simple blending method.

## 2. Experimental

### 2.1 Materials

MWCNTs (OD: 8–15 nm, length: 0.5–2 μm, purity >98%) and MWCNT-OHs (OD: 8–15 nm, length: 0.5–2 μm, purity >98%, –OH content: 3.06 wt%) were bought from Chengdu Organic Chemicals Co., Ltd. Chinese Academy of Sciences. Pyromellitic dianhydride (PMDA) supplied by Sinopharm Chemical Reagent Beijing Co. Ltd was dried in vacuum at 200 °C for 10 h prior to use. 4,4′-Diaminodiphenyl ether (4,4′-ODA) also supplied by Sinopharm Chemical Reagent Beijing Co., Ltd was dried in vacuum at 80 °C for 10 h prior to use. (3-Aminopropyl)triethoxysilane (APTES) supplied by Aladdin Reagent Co., Ltd was used without further purification. *N*,*N*-Dimethylacetamide (DMAc) was purified by vacuum distillation and stored in a bottle in the presence of 4 Å molecular sieves prior to use.

### 2.2 Measurements

FTIR spectra were recorded on a Nicolet iS10 spectrometer at a resolution of 2 cm^−1^ in the range of 400–4000 cm^−1^ with reflection mode. Dynamic Mechanical Analysis (DMA) was performed with a TA instrument (DMA Q800) at the heating rate of 5 °C min^−1^ and a load frequency of 1 Hz in the film tension geometry and *T*_g_ was regarded as the peak temperature of tan *δ* curves. Thermogravimetric analysis (TGA) was performed with the TA instrument 2050, with a thermal heating rate of 10 °C min^−1^ in nitrogen or air atmosphere. The mechanical properties of the samples were studied at room temperature by a Shimadzu AG-I universal testing apparatus with a crosshead speed of 2 mm min^−1^. Measurements were obtained at 25 °C with film specimens (about 50 μm thick, 6 mm wide and 40 mm long). The cross-section morphology of films was observed by Scanning Electron Microscopy (SEM, NOVA NANOSEM 450, England). The films were fractured in liquid nitrogen and coated with gold prior to test. Thermal conductivity measurements were performed at 25 °C by thermal conductivity instrument of TC 3000 series based on ASTM D5930 Standard Test Method for Thermal Conductivity of Plastics by means of a Transient Line Source Technique. Thermal conductivity *K* (W m^−1^ K^−1^) was calculated by the following equation:
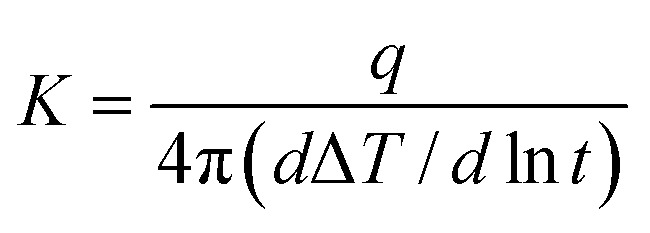
Here, *q* represents the heat conducted per unit length of the wire, Δ*T* represents the temperature changes in the wire and *t* represents the measuring time.

### 2.3 Material preparations

#### 2.3.1 Synthesizing MWCNT/PIs *via* blending method

Samples with different MWCNT contents (0%, 5%, and 10%) in polyimide were synthesized *via* the blending method and designated as PI, 5% MWCNT/PI, and 10% MWCNT/PI, respectively. The preparation of 5% MWCNT/PI was used as a representative to illustrate the detailed synthetic procedure. First, 0.2202 g MWCNTs and 25 g DMAc were added into a three-neck flask and then, the mixture was subjected to ultrasonic dispersion at room temperature for 3 h. Subsequently, ODA (10 mmol, 2.002 g), PMDA (10 mmol, 2.181 g), and 14.6 g DMAc were added into the three-neck flask. The reaction mixture was slowly stirred for 24 h. Next, the mixture was casted on a glass plate, followed by a preheating program (60 °C/10 h, 80 °C/2 h, 100 °C/2 h, 120 °C/2 h) and an imidization procedure under vacuum (200 °C/1 h, 250 °C/1 h, and 300 °C/1 h) to produce the 5% MWCNT/PI film.

#### 2.3.2 Preparing MWCNT-*g*-PIs by *in situ* grafting method (Scheme 1)

The different MWCNT contents (0%, 5%, and 10%) were grafted on polyimide *via* the *in situ* synthesis method and the corresponding samples were named *g*-PI, 5% MWCNT-*g*-PI, and 10% MWCNT-*g*-PI. The preparation of 5% MWCNT-*g*-PI was used as a representative to illustrate the detailed synthetic procedure. First, 0.2204 g MWCNT-OH and 25 g DMAc were added into a three-neck flask and then, the mixture was subjected to ultrasonic dispersion at room temperature for 3 h. Subsequently, ODA (9.8 mmol, 1.962 g), PMDA (10 mmol, 2.181 g), and 14.7 g DMAc were added into the three-neck flask. The reaction mixture was slowly stirred for 2 h. At last, APTES (0.2 mmol, 0.0443 g) was introduced into the system, and the system underwent polymerization for 24 h. Then, the mixture was casted on a glass plate, followed by a preheating program (60 °C/10 h, 80 °C/2 h, 100 °C/2 h, 120 °C/2 h) and an imidization procedure under vacuum (200 °C/1 h, 250 °C/1 h, and 300 °C/1 h) to produce the 5% MWCNT-*g*-PI film.

**Scheme 1:**
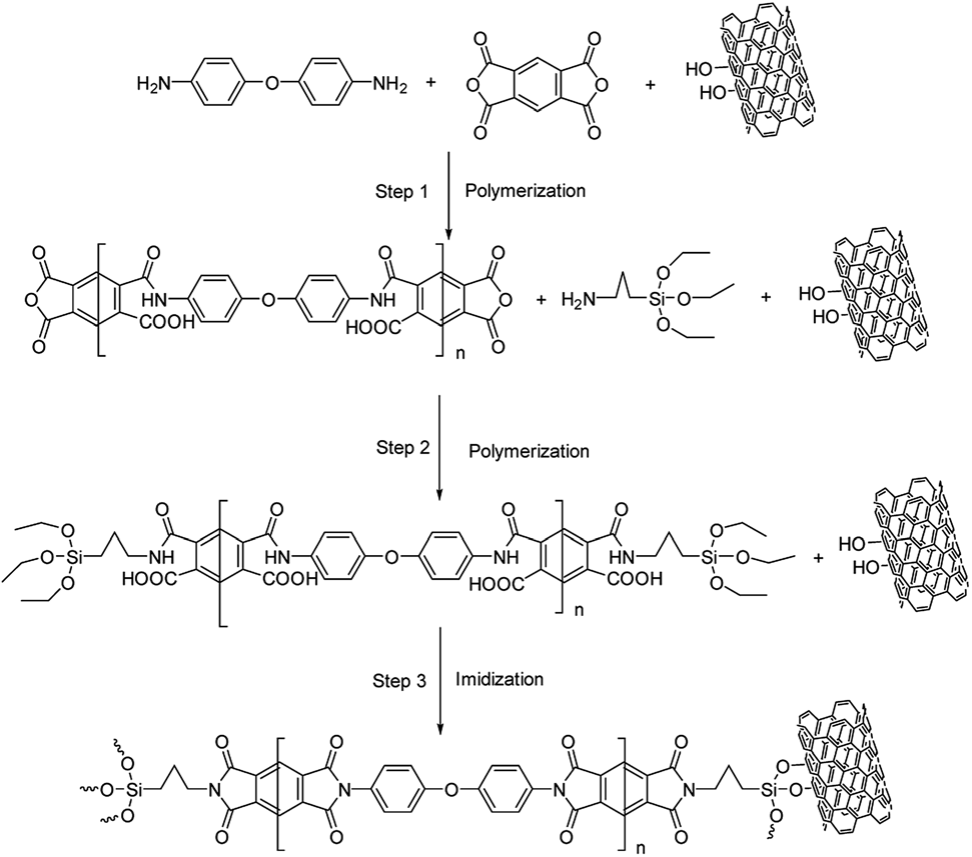
The preparation process of MWCNT-*g*-PIs.

## 3. Results and discussion

### 3.1 Characterization of MWCNT/PIs and MWCNT-*g*-PIs

**Fig. 1:**
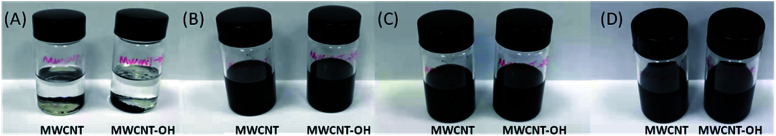
The dispersed state of carbon nanotubes in 1 mg mL^−1^ DMAc: (A) before sonication; (B) after sonication for 3 h; (C) 12 h after sonication; (D) 24 h after sonication.

Fig. 1 demonstrates the dispersed state of carbon nanotubes in DMAc. Fig. 1(B) shows the dispersed state after sonicating for 3 h; this indicated that MWCNTs and MWCNT-OH were equally dispersed in DMAc and stood for 12 h and 24 h, respectively, after sonication without sedimenting towards the bottom of the bottle evidently (Fig. 1(C) and (D)). Hence, DMAc was selected as the solvent to disperse carbon nanotubes and synthesize MWCNT/PIs and MWCNT-*g*-PIs.

**Fig. 2:**
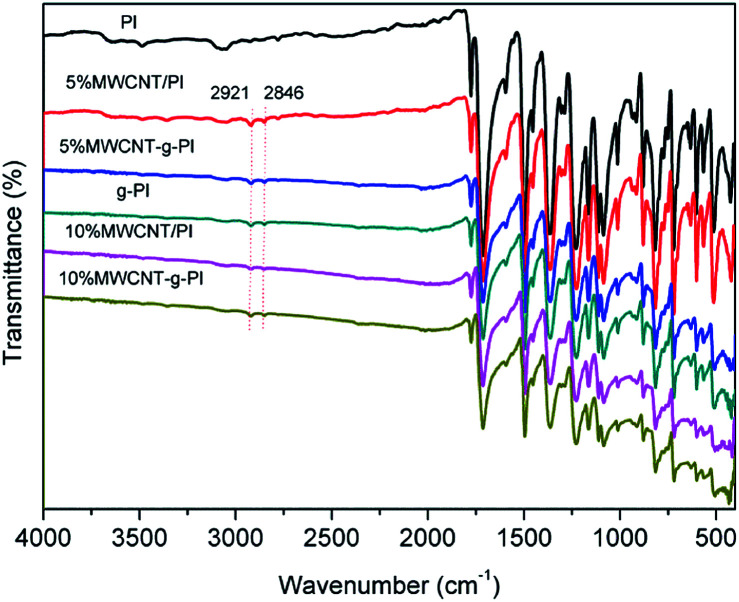
FT-IR spectra of MWCNT/PIs and MWCNT-*g*-PIs.

**Fig. 3:**
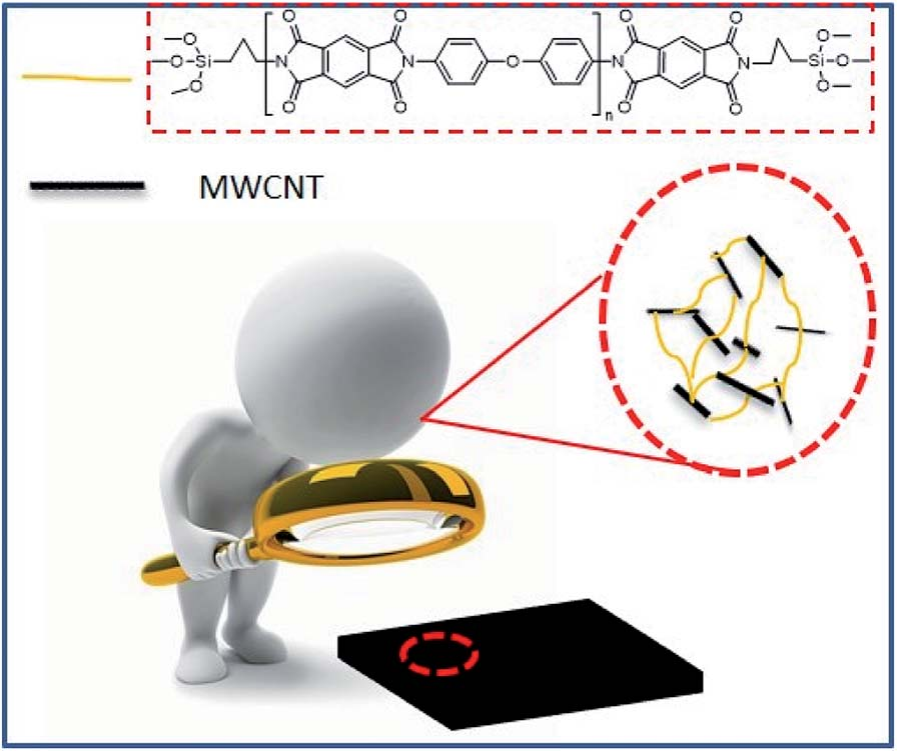
Illustration of the interaction between MWCNTs and polyimide in MWCNT-*g*-PIs.

The chemical structures of MWCNT-*g*-PIs were characterized by FT-IR spectroscopy. Fig. 2 demonstrates the FT-IR spectra for MWCNT/PIs and MWCNT-*g*-PIs. All the samples exhibited characteristic imide absorptions at around 1776 cm^−1^ (asymmetrical C=O stretching), 1714 cm^−1^ (symmetrical C=O stretching), and 1366 cm^−1^ (C–N stretching). The spectra of MWCNT-*g*-PIs show the asymmetrical and symmetrical stretching vibrations of –CH_2_ at 2921 cm^−1^ and 2846 cm^−1^, respectively. These vibrations belonged to APTES and carbon nanotubes, and no existence of the characteristic absorption bands of the –NH_2_ and –OH groups proved the successful grafting of polyimide chains on carbon nanotubes. The interaction between MWCNT and PI in MWCNT-*g*-PIs *via* coupling is illustrated in Fig. 3.

**Fig. 4:**
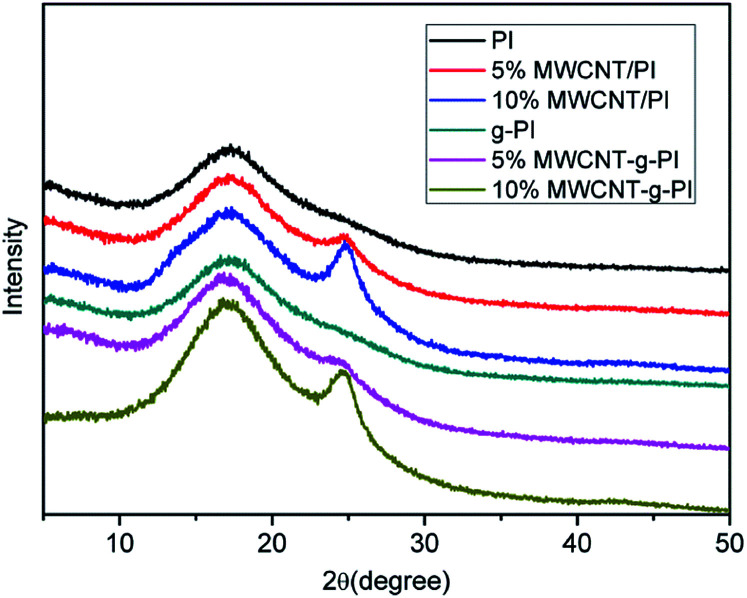
XRD spectra of MWCNT/PIs and MWCNT-*g*-PIs.

Fig. 4 shows the wide-angle X-ray diffraction (XRD) curves of MWCNT/PIs and MWCNT-*g*-PIs. PI and *g*-PI only exhibited a diffuse peak at 2*θ* = 17.5°, whereas 5% and 10% MWCNT/PIs and MWCNT-*g*-PIs exhibited two diffuse peaks at 2*θ* = 17.3° and 24.9°, respectively. A small diffuse peak at 2*θ* = 24.9° was observed in the diffraction curves of 5% and 10% MWCNT/PIs and MWCNT-*g*-PIs, indicating that the carbon nanotubes were successfully incorporated into the polyimide matrix.

### 3.2 Thermal properties of MWCNT/PIs and MWCNT-*g*-PIs

**Fig. 5:**
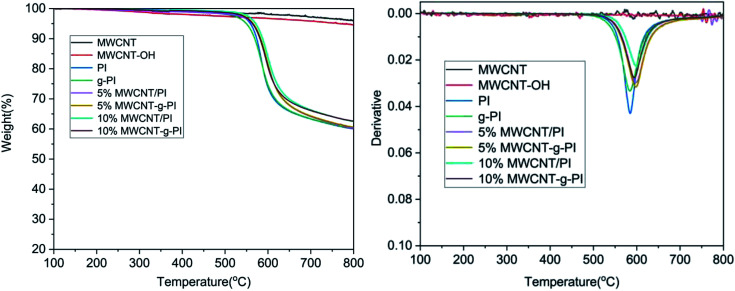
TGA and DTG curves of MWCNTs, MWCNT-OH, MWCNT/PIs and MWCNT-*g*-PIs in N_2_.

**Fig. 6:**
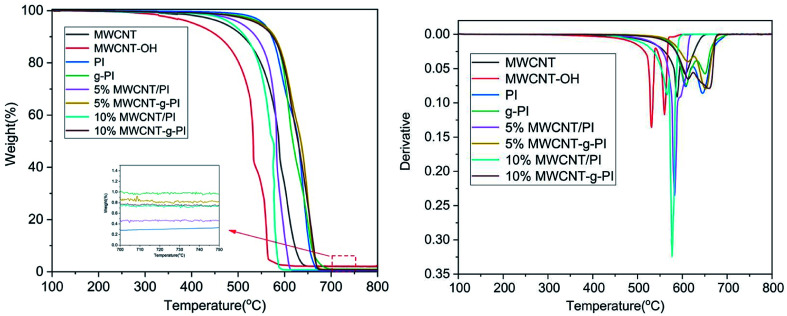
TGA and DTG curves of MWCNTs, MWCNT-OH, MWCNT/PIs and MWCNT-*g*-PIs in air.

Fig. 5 and 6 represent the thermal stability of MWCNT/PIs and MWCNT-*g*-PIs investigated by TGA under N_2_ and air atmospheres at the heating rate of 10 °C min^−1^; the results are listed in Table 1. The *T*_5%_ and *T*_10%_ values of PI were 559 °C and 573 °C under N_2_ atmosphere, respectively. Compared with the values for PI, the *T*_5%_ and *T*_10%_ of *g*-PI decreased slightly under N_2_ atmosphere; the values were 549 °C and 569 °C, respectively. However, the addition of carbon nanotubes improved *T*_5%_ and *T*_10%_ under N_2_ atmosphere irrespective of whether by blending or grafting. By the thermal degradation curves of MWCNTs and MWCNT-OH under N_2_ atmosphere, we can infer that MWCNTs and MWCNT-OH have better thermal stability than PI, which results in the enhancement of *T*_5%_ and *T*_10%_ of the materials. The residual weight retentions at 800 °C also improved under N_2_ atmosphere; the values for 10% MWCNT/PI and 10% MWCNT-*g*-PI were 62.5% and 62.6%, respectively. In contrast to the values for PI, *T*_5%_ and *T*_10%_ had a marked decrease under air atmosphere for the materials prepared by the blending method. From the DTG curves of MWCNT/PIs in air, we can infer that the degradation of MWCNTs at a high-temperature stage is the main reason for the above-mentioned phenomenon. However, *T*_5%_ and *T*_10%_ had a marked increase under air atmosphere for the materials prepared by the grafting method than the results obtained for the blending method. After grafting, MWCNTs were tightly wrapped by polyimide chains owing to the covalent bond linkage between MWCNTs and polyimide chains, which strengthened the interfacial interaction and thus, the MWCNT degradation was delayed. The heat-resistance index (*T*_HRI_) was calculated;^11,20^ the results are listed in Table 1. In N_2_, the *T*_HRI_ values of MWCNT/PIs and MWCNT-*g*-PIs increased after the addition of MWCNTs. Under air atmosphere, the *T*_HRI_ of MWCNT/PIs decreased after the addition of MWCNTs, but *T*_HRI_ of the MWCNT-*g*-PIs brought into correspondence with that of pure PI. In short, MWCNT/PIs and MWCNT-*g*-PIs exhibited good thermal stability in N_2_. However, MWCNT-*g*-PIs possessed better thermal stability than MWCNT/PIs in air. Nevertheless, the reduction in thermal stability for MWCNT/PIs in air was retained at an acceptable degree.

**Table 1:** Thermal properties of MWCNT/PIs and MWCNT-*g*-PIs

Sample codes	*T*_g_ a (°C)	*T*_5%_ b (°C)		*T*_10%_ (°C)		*T*_30%_ (°C)		*T*_HRI_ c (°C)		*R*_w_ d (%)
		N_2_	Air	N_2_	Air	N_2_	Air	N_2_	Air	
PI	399	559	559	573	573	614	600	290	286	60.1
5% MWCNT/PI	400	563	507	582	539	634	575	297	268	60.2
10% MWCNT/PI	398	571	486	587	516	649	557	303	259	62.5
*g*-PI	412	549	551	569	577	617	603	289	285	60.2
5% MWCNT-*g*-PI	404	565	549	583	578	635	611	297	287	60.6
10% MWCNT-*g*-PI	401	564	543	581	575	642	609	299	286	62.6

a Measured by DMA at a heating rate of 5 °C min^−1^.

b 5% weight loss temperature (*T*_5%_) and 10% weight loss (*T*_10%_) temperature measured by TGA.

c Heat-resistance index (*T*_HRI_) was calculated by the equation *T*_HRI_ = 0.49 × [*T*_5%_ + 0.6 × (*T*_30%_ − *T*_5%_)].

d Residual weight retention at 800 °C.

**Fig. 7:**
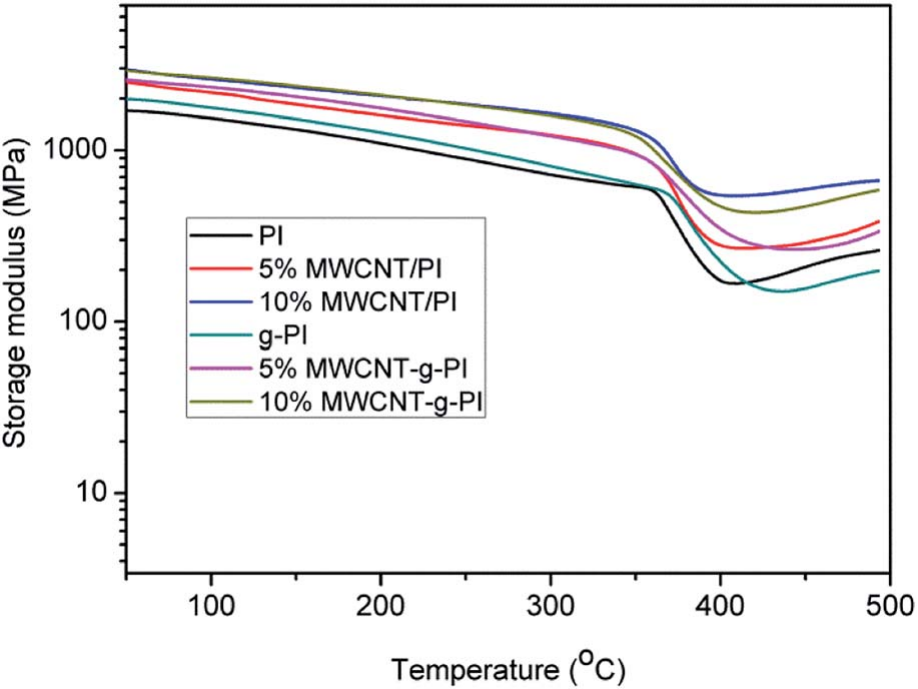
Storage modulus curves of MWCNT/PIs and MWCNT-*g*-PIs.

**Fig. 8:**
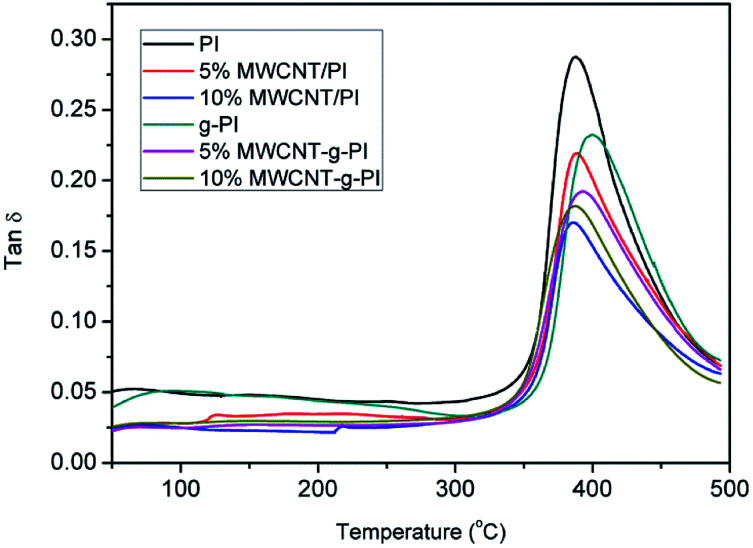
tan *δ* curves of MWCNT/PIs and MWCNT-*g*-PIs.

The dynamic mechanical analyses of MWCNT/PIs and MWCNT-*g*-PIs are shown in Fig. 7 and 8. The storage modulus retentions of MWCNT/PIs and MWCNT-*g*-PIs at 200 °C and 300 °C were analysed and listed in Table 2. All the samples had good storage modulus retention at a high-temperature stage. The storage modulus retentions were greater than 63% at 200 °C and 45% at 300 °C. Meanwhile, the glass transition temperature (*T*_g_) was analysed; it was determined by the peak temperature of the tan *δ* curves and listed in Table 1. *T*_g_ is possibly determined by two competitive factors: the free volume and the steric effect.^21,22^ In the MWCNT-*g*-PI system, *T*_g_ shows a decreasing trend with the increase in the loading amount of MWCNTs. The polyimide chains grafted on the MWCNT surfaces disrupted the ordered chain structure of the polyimides and resulted in the increase in free volume. However, the *T*_g_ values of all the samples were greater than 399 °C.

**Table 2:** Storage modulus retention of MWCNT/PIs and MWCNT-*g*-PIs decided by storage modulus curves^a^

Sample codes	Storage modulus retention (%)	
	200 °C	300 °C
PI	68.0	53.4
5% MWCNT/PI	70.2	52.4
10% MWCNT/PI	72.4	53.4
*g*-PI	63.9	45.8
5% MWCNT-*g*-PI	68.3	50.8
10% MWCNT-*g*-PI	67.1	50.1

a
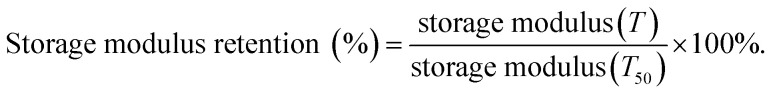

### 3.3 Mechanical properties of MWCNT/PIs and MWCNT-*g*-PIs

For the nanocomposites, the mechanical property is affected by many factors, such as the polymer matrix, loading amount of inorganic nanofillers, dispersion in the polymer matrix and interfacial interaction.^23^ Based on the several aspects mentioned above, the mechanical properties of MWCNT/PIs and MWCNT-*g*-PIs were discussed. The tensile strength, tensile modulus and elongation at break results of MWCNT/PIs and MWCNT-*g*-PIs are summarized in Table 3. The tensile strength, tensile modulus and elongation at break of PI were 129 MPa, 2.39 GPa and 57.5%, respectively. PI showed good mechanical properties. Compared with the results for PI, the tensile strength and tensile modulus of *g*-PI had a slight increase because of the existence of crosslinking points by the self-polycondensation of the coupling agent at the ending of the polyimide chains, which was also responsible for the decrease in the elongation at break of *g*-PI from 57.5% to 48.6%. Subsequently, the mechanical properties of MWCNT-*g*-PIs with different loading amounts were analysed. 5% and 10% MWCNT-*g*-PIs maintained the high tensile strength of PI. The tensile modulus increased up to 2.59 GPa on increasing the MWCNT loading. The elongation at break of MWCNT-*g*-PIs exhibited a reducing trend but was still more than 36%. The reduction in the elongation at break in our system was retained at an acceptable degree. The covalent bond linkage between MWCNTs and polyimide chains promoted the well-distributed dispersion of MWCNTs in polyimides and strengthened the interfacial interaction between MWCNTs and polyimide chains. Hence, MWCNT-*g*-PIs showed good mechanical properties. The mechanical properties of the sample prepared by the simple blending method (MWCNT/PIs) were also investigated. In this research, a short carbon nanotube (*L*/*d* = 250) was selected, which could be easily dispersed in a polymer matrix and lead to the existence of π–π interactions between the carbon nanotubes and benzene rings in polyimide chains. Thus, MWCNT/PIs also exhibited good mechanical properties.

**Table 3:** Mechanical properties of MWCNT/PIs and MWCNT-*g*-PIs

Sample codes	*T*_S_ a (MPa)	*T*_M_ b (GPa)	*E*_B_ c (%)
PI	129	2.38	57.5
5% MWCNT/PI	132	2.39	53.6
10% MWCNT/PI	128	2.70	41.8
*g*-PI	132	2.39	48.6
5% MWCNT-*g*-PI	124	2.41	36.6
10% MWCNT-*g*-PI	129	2.59	36.1

a *T*_S_, tensile strength.

b *T*_M_, tensile modulus.

c *E*_B_, elongation at break.

### 3.4 Morphology of MWCNT/PIs and MWCNT-*g*-PIs

**Fig. 9:**
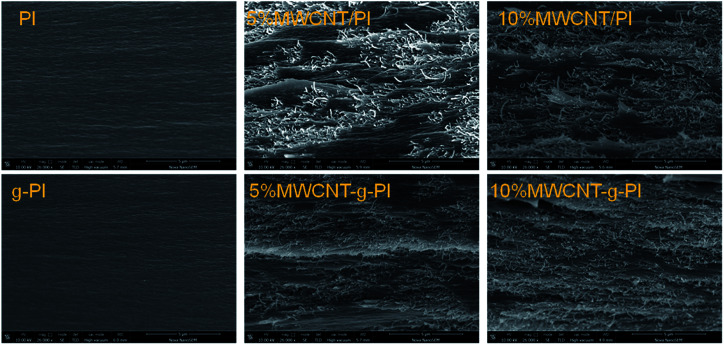
Cross-section morphology of MWCNT/PIs and MWCNT-*g*-PIs.

Fig. 9 exhibits the SEM images of MWCNT/PIs and MWCNT-*g*-PIs. In Fig. 9, it can also be noticed that the MWCNTs disperse more homogeneously in MWCNT-*g*-PIs than in MWCNT/PIs due to covalent bond linkage, strengthening the interfacial interaction between MWCNTs and the polyimide matrix. A small portion of agglomerated MWCNTs can be seen in the 5% and 10% MWCNT/PI composites. This is one of the key factors that can affect the thermal conductivity of the resulting composites. Apparently, a higher filler content is required to form “thermal conductive pathways” when the fillers agglomerate in the polymer matrix. A good dispersion of MWCNTs in polyimides may contribute to the improvement in thermal conductivity.

### 3.5 Thermal conductivity of MWCNT/PIs and MWCNT-*g*-PIs

**Fig. 10:**
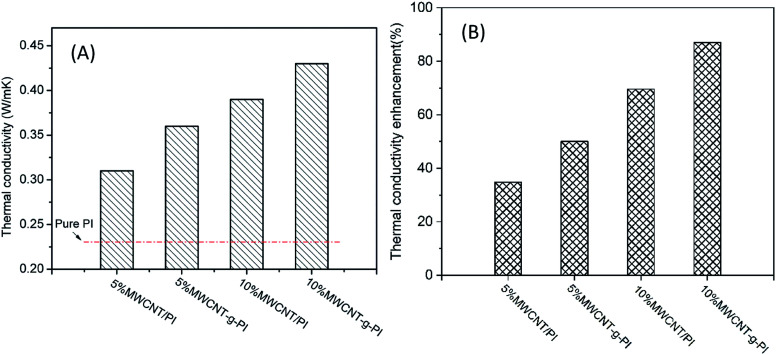
Thermal conductivity (A) and thermal conductivity enhancement (B) of MWCNT/PIs and MWCNT-*g*-PIs.

The thermal conductivity properties of the MWCNT/PIs and MWCNT-*g*-PI composites are shown in Fig. 9. The increasing MWCNT loading enhanced the thermal conductivity of MWCNT/PIs and MWCNT-*g*-PIs because more and more MWCNTs participated in forming “thermal conductive pathways”. However, the thermal conductivity of MWCNT-*g*-PIs increased faster than that of MWCNT/PIs at the same loading. The thermal conductivity of 10% MWCNT/PIs improved by 69.6% than that of pure PI. The thermal conductivity of 10% MWCNT-*g*-PIs increased by 87.0% than that of pure PI (Fig. 10). The well-distributed dispersion of MWCNTs in polyimides can be boosted to form “thermal conductive pathways” at the same loading, and strengthening the interfacial interaction between MWCNTs and polyimide chains by covalent bond linkage can decrease the interfacial thermal resistance (*R*_C_) between nanotubes and the polymer matrix.

The interfacial thermal resistance (*R*_C_) was calculated by the Maxwell-Garnett-type effective medium approach (EMA) in our research as follows:^24^1
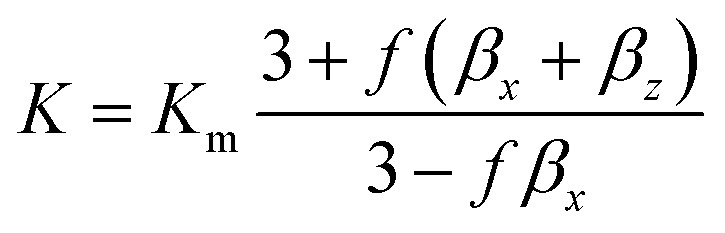
2
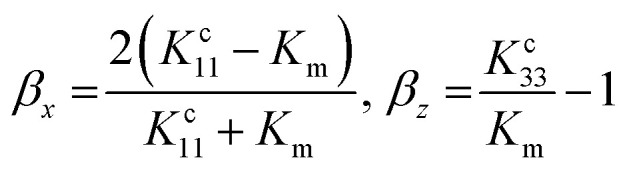
Here, *f* is the volume fraction of the carbon nanotubes, *K*_m_ represents the thermal conductivity of PI (0.23 W m^−1^ K^−1^), *K*_c_ represents the thermal conductivity of MWCNTs (3000 W m^−1^ K^−1^), *K*^c^_11_ represents the equivalent thermal conductivities along transverse and *K*^c^_33_ represents the longitudinal axes of the composite unit cell. *K*^c^_11_and *K*^c^_33_ can be expressed as follows:3
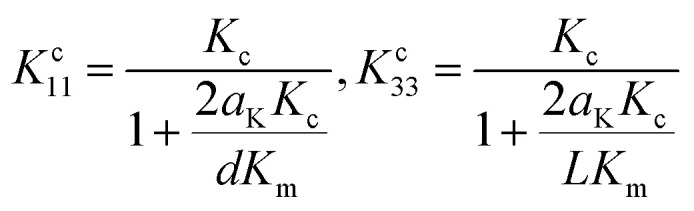
Here, *d* represents the diameter of the carbon nanotubes (*d* = 8 nm) and *L* represents the length of the carbon nanotubes (*L* = 2 μm); *a*_K_ is the so-called Kapitza radius, which is expressed as follows:

4 *a*_K_ = *R*_C_*K*_m_

According to the above-mentioned equations, the interfacial thermal resistance (*R*_C_) of 3.0 × 10^−8^ m^2^ K W^−1^ for the 10% MWCNT/PI composite and 2.5 × 10^−8^ m^2^ K W^−1^ for the 10% MWCNT-*g*-PI composite was observed. In contrast to the value for 10% MWCNT/PI, the interfacial thermal resistance (*R*_C_) of 10% MWCNT-*g*-PI decreased by 16.7%, which was consistent with the results we expected. Strengthening the interfacial interaction between MWCNTs and polyimide chains is necessary to reduce the interfacial thermal resistance (*R*_C_) and then improve the thermal conductivity.

The thermal conductivity data of MWCNT-graft-polyimides were compared with that reported for MWCNT/polymer composites in the current literature; the results are listed in Table 4. The data showed that the thermal conductivity of MWCNT-graft-polyimides was higher than that of most MWCNT/polymer composites reported previously. Compared with the thermal conductivity of 10% MWCNT/PI, the thermal conductivity of 10% MWCNT-*g*-PI increased by 10.3%.

**Table 4:** Thermal conductivity of MWCNT/polymer composites listed in literature compared with the results of this work

Composites	Loading (wt%)	Thermal conductivity (W m^−1^ K^−1^)	Year and ref
MWCNT/poly(ether ether ketone)	17	0.70	2009 (ref. 25)
MWCNT/expoxy	10	0.35	2012 (ref. 16)
MWCNT/phenoxy	10	0.42	2013 (ref. 26)
MWCNT/poly(ether sulfone)	10	0.35	2013 (ref. 26)
MWCNT/polyamide-6	4	0.28	2009 (ref. 27)
MWCNT/polyimide	3	0.25	2019 (ref. 3)
MWCNT/expoxy	2	0.25	2019 (ref. 5)
MWCNT-graft-polyimides	5	0.36	This work
	10	0.43	

## 4. Conclusions

In this paper, it was found that MWCNT-*g*-PIs possessed higher thermal conductivity than MWCNT/PIs. In contrast to the interfacial thermal resistance of 10% MWCNT/PIs, the interfacial thermal resistance of 10% MWCNT-*g*-PI decreased by 16.7%. Meanwhile, MWCNT/PIs and MWCNT-*g*-PIs showed good thermal and mechanical properties. The *T*_g_ values of all the samples were greater than 399 °C. The thermal stability of 5% and 10% MWCNT-*g*-PIs in air was enhanced compared to that for 5% and 10% MWCNT/PIs. The storage modulus retentions were greater than 63% at 200 °C and 45% at 300 °C. 5% and 10% MWCNT-*g*-PIs maintained the high tensile strength of PI. The tensile modulus increased up to 2.59 GPa with the increase in the MWCNT loading. This work provides a guideline for improving the thermal conductivity of polyimides effectively at relatively low loadings.

## Conflicts of interest

There are no conflicts to declare.

## Supplementary Material
